# Structural Basis for TGF-β Mimetic Peptide-Induced Signaling Activation Through Molecular Dynamics Simulations

**DOI:** 10.3390/ijms27010022

**Published:** 2025-12-19

**Authors:** Chun Chen, Jingsong Ai, Junhui Huang, Xiaobin Li, Yiting Wang, Mingjie Tong, Xinshan Xie, Qiuling Xie, Sheng Xiong

**Affiliations:** 1 College of Life Science and Technology, Jinan University, Guangzhou 510632, China; chenchun_jnu@163.com (C.C.); huangjunhui@stu2024.jnu.edu.cn (J.H.); wyt2010@126.com (Y.W.); tongmingjie0213@163.com (M.T.); 17314973843@163.com (X.X.); 2 MOE Key Laboratory for Nonequilibrium Synthesis and Modulation of Condensed Matter, School of Physics, Xi’an Jiaotong University, Xi’an 710049, China; aijingsong@stu.xjtu.edu.cn

**Keywords:** transforming growth factor-β (TGF-β), TGF-β-mimetic peptide, protein dynamics, molecular dynamics (MD) simulation, AlphaFold3, signal transduction

## Abstract

Transforming growth factor-β (TGF-β) mimetic peptides offer significant therapeutic potential due to their superior pharmacological properties over the native cytokine. Our previous work identified two such peptides, TB1 and TB2, which bind to the type II TGF-β receptor (TβRII) yet elicit distinct cellular responses. To uncover the mechanistic basis for the functional divergence, we employed integrated molecular dynamics (MD) simulations with the AlphaFold3-predicted structures. Our analytical results indicated that TB2 stabilizes a dynamic complex with TβRII and is predicted to facilitate type I receptor (TβRI) engagement possibly involving a critical hydrogen bond between TB2-Gly11 and TβRI-Phe60. The resulting trimeric assembly (TB2–TβRII–TβRI) exhibits a higher relative binding affinity (−67.76 ± 7.70 kcal/mol) and structural stability. In contrast, the TB1–TβRII complex fails to productively engage TβRI. These computational results were experimentally validated. Western blot analysis confirmed that TB2, but not TB1, activates the canonical TGF-β/Smad pathway by enhancing the expression and phosphorylation of Smad3. This study will elucidate the dynamic structural basis for the activity of TGF-β mimetic peptides and suggest TB2 as a promising lead candidate for the rational design of tissue-regenerative therapeutics.

## 1. Introduction

Transforming growth factor-beta (TGF-β) is a critical cytokine that regulates diverse cellular processes, including proliferation, differentiation, apoptosis, migration, and immune responses [1,2,3,4]. Its activity is mediated through binding to transmembrane receptors, primarily the high-affinity TGF-β type II receptor (TβRII) and the low-affinity TGF-β type I receptor (TβRI) [5,6,7]. Due to its biological activity, TGF-β holds considerable therapeutic potential in chronic wound healing, osteoarthritis treatment, and tissue repair [8,9,10,11,12,13,14]. During the regeneration of tissues, TGF-β promotes repair by stimulating collagen synthesis and extracellular matrix (ECM) deposition [13,15].

**Scheme 1:**
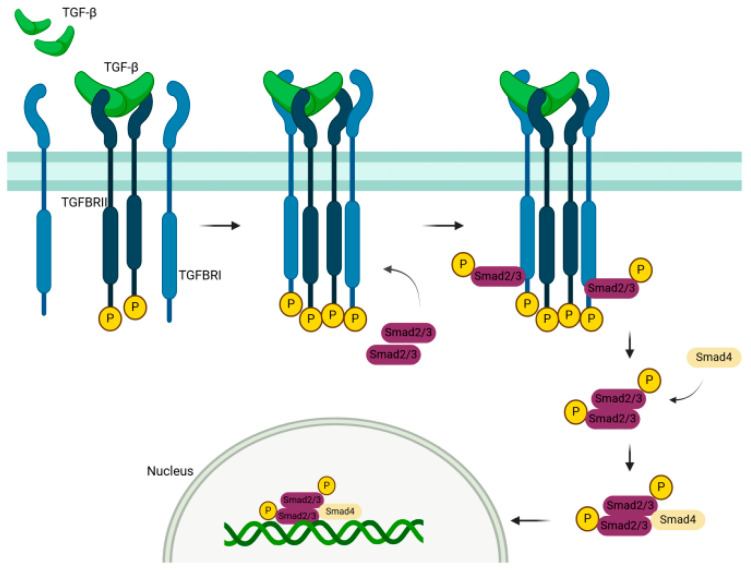
The canonical TGF-β signaling pathway via TGF-β/Smad3. TGF-β initially binds to TGFBRII, which subsequently recruits TGFBRI to form a receptor complex. The activated TGFBRI phosphorylates the C-terminal region of Smad2/3. Phosphorylated Smad2/3 then associates with Smad4 to form a transcription factor complex that regulates downstream gene expression.

TGF-β signaling is mediated by both Smad-dependent and Smad-independent pathways [1,16]. In the canonical Smad-dependent pathway, TGF-β ligand binding to TβRII leads to the recruitment of TβRI, which subsequently phosphorylates Smad2/3 at their C-terminal domains [17,18]. Phosphorylated Smad2/3 form a complex with Smad4 and translocate into the nucleus to regulate the expression of target genes [19,20] (Scheme 1). In contrast, Smad-independent pathways trigger signaling cascades such as MAPK (ERK/JNK/p38) and PI3K-Akt, which modulate cytoskeletal dynamics, apoptosis, and immune responses [2,21,22,23]. The interplay between these pathways enables TGF-β signaling to respond dynamically to changes in the cellular microenvironment [2].

Current drug development targeting the TGF-β pathway exhibits a distinct trend toward inhibitors—such as neutralizing antibodies, ligand traps, and kinase inhibitors—which aim to block pathway hyperactivation in cancer and fibrosis [2,24]. In contrast, therapeutic agonists capable of promoting tissue repair are scarce. While recombinant proteins like Avotermin (rhTGF-β3) have shown promise in clinical trials for scar improvement [25], their widespread application faces challenges inherent to protein therapeutics, such as high production costs, complex production processes, immunogenicity and poor tissue penetration [26]. Peptide-based therapeutics have attracted increasing interest due to their stability, ease of synthesis, and efficient tissue delivery [27,28,29,30]. To develop peptides that mimic the biological functions of TGF-β and activate the TGF-β/Smad3 pathway, our laboratory employed phage display technology [31,32] targeting the extracellular domain of TβRII, which led to the identification of two peptides: TB1 (LDSQAPMGRINH) and TB2 (KLHHHLHVPRGP). In vitro experiments demonstrated that the two peptides bind to TβRII but exhibit distinct biological activities (these data are derived from a separate, ongoing study): TB1 promotes proliferation of 3T3 cells, whereas TB2 not only stimulates 3T3 cell proliferation but also enhances the expression of type I and type III collagen. However, the molecular mechanisms and binding profiles for these different functions remain unclear. Elucidating these mechanisms will benefit the development of tissue repair by the peptide-based therapeutics.

In this study, we combined AlphaFold3 [33,34,35] predictions with molecular dynamics (MD) simulations to investigate the structural basis of TB1 and TB2 binding to TβRII and the structural basis for their capacity to recruit TβRI. Although AlphaFold3 significantly improves the accuracy of protein–ligand interaction modeling [35], it primarily yields static structural snapshots. Biological molecules exert their functions in vivo through dynamic behaviors rather than static structures [36]. To capture the conformational fluctuations essential for receptor activation, we performed extensive MD simulations [37,38,39] on both dimeric (TB1–TβRII, TB2–TβRII) and trimeric (TB1–TβRII–TβRI, TB2–TβRII–TβRI) complexes. We further employed MM-PBSA [40,41,42] calculations to evaluate binding free energies and residue-specific contributions. Finally, Western blot analysis was conducted to experimentally validate the activation of the TGF-β/Smad pathway by the peptides. Our integrated computational and experimental approach reveals that TB2 stabilizes a dynamic complex with TβRII and actively recruits TβRI, a process facilitated by a critical hydrogen bond between TB2-Gly11 and TβRI-Phe60. In contrast, TB1 is predicted to show limited interaction with TβRI, which may partly account for its weaker downstream signaling activity. These findings not only elucidate the mechanistic basis for the functional divergence between TB1 and TB2 but also highlight TB2 as a promising lead for the rational design of TGF-β-based regenerative therapeutics.

## 2. Results

### 2.1. AlphaFold3-Driven Modeling

**Figure 1:**
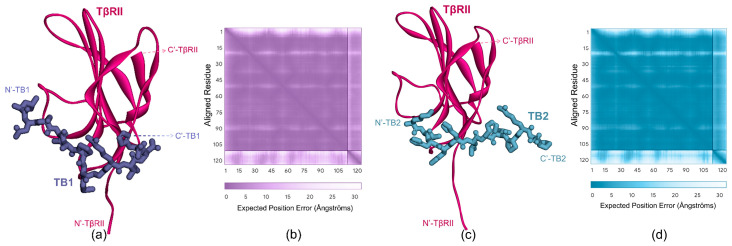
AlphaFold3-predicted structures and the confidence matrix plots of TB1–TβRII and TB2–TβRII. (**a**) Predicted dimeric structure of TB1–TβRII using AlphaFold3. (**b**) Expected Position Error (EPE) of TB1–TβRII. (**c**) Predicted dimeric structure of TB2–TβRII using AlphaFold3. (**d**) Expected Position Error (EPE) of TB2–TβRII. The color gradient indicates the expected position error (in Å) for each residue. Chain borders were visualized as thin black solid lines.

AlphaFold3 was employed to construct the dimeric complexes TB1-TβRII and TB2-TβRII. As shown in Figure 1, in the two generated structures (Figure 1a,c), most regions of the expected position error (EPE) matrix plots (Figure 1b,d) displayed a relatively uniform dark tone, indicating that the expected positional errors of these residues were small and that the model’s structural predictions for these regions were relatively accurate. For the TB1 model, the pTM score was 0.88 and the ipTM score was 0.60, while for the TB2 model, the pTM score was 0.86 and the ipTM score was 0.46. From the results, the pTM scores are all above 0.8, indicating that the overall structures are relatively reasonable [34,43]. However, for the ipTM score, the TB2 model exhibited a lower ipTM of 0.46, suggesting potential imperfections at the interaction interface. A low ipTM does not inherently invalidate the model; for instance, the TB2 peptide interacts with TβRII primarily through partial N-terminal residues, resulting in fewer atomic contacts and a smaller interface area compared to TB1.

**Figure 2:**
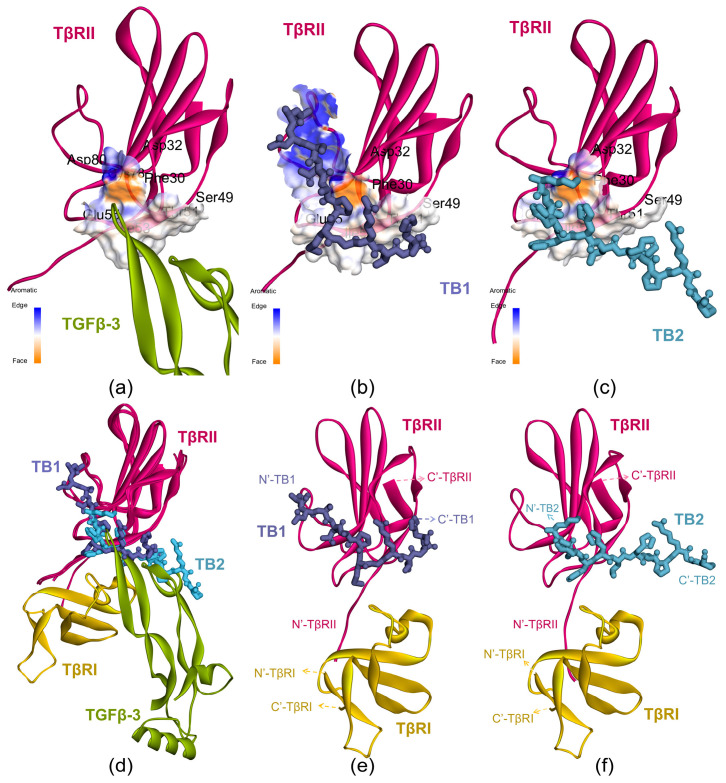
Interaction surfaces of TB1, TB2, and TβRII, and homology modeling of TB1–TβRII–TβRI and TB2–TβRII–TβRI. (**a**) Interaction surface between TGF-β3 and TβRII, derived from the X-ray crystallographic structure of the TGF-β–receptor complex (PDB: 2PJY). (**b**) Interaction surface of TB1 with TβRII. (**c**) Interaction surface of TB2 with TβRII. (**d**) Structural superposition of AlphaFold3-predicted TB1–TβRII and TB2–TβRII with the X-ray-resolved TGF-β multimeric signaling complex (PDB: 2PJY). The TβRII chains in all three structures are highly consistent. TB1 and TB2 share partially overlapping epitopes on TβRII, whereas the epitope of TB2 binding resembles that of TGF-β3 more closely. (**e**) Homology model of TB1–TβRII–TβRI based on TB1–TβRII, the crystal structures of TβRI (PDB: 2L5S) and TβRII (PDB: 1PLO), and the TGF-β–receptor complex (PDB: 2PJY). (**f**) Homology model of TB2–TβRII–TβRI based on TB2–TβRII, the crystal structures of TβRI (PDB: 2L5S) and TβRII (PDB: 1PLO), and the TGF-β–receptor complex (PDB: 2PJY).

Structural alignment of the predicted complexes with the X-ray crystallographic structure of the TGF-β receptor complex (PDB: 2PJY) revealed partial overlap of the epitopes where TB1 and TB2 interact with TβRII. Notably, the predicted TB2 binding site on TβRII appears to partially resemble that of TGF-β3. In the TB2-TβRII complex, residues of TβRII located within 5 Å of TB2 included Leu27–Asp32 and Ser49–Glu55 (residue numbering based on PDB 2PJY, with TβRII sequence spanning Ala19–Phe126). This region substantially overlapped with the binding domain of TGF-β3 on TβRII in the 2PJY structure. In contrast, in the TB1-TβRII system, residues within 5 Å of TB1 included Leu27–Asp32, Ser49–Glu55, Val77–Asp80, and Pro84–Asp87. The predicted similarity of TB1 and TB2 binding sites to those of TGF-β3 may account for their TGF-β3-like biological activities in promoting cell proliferation (Figure 2a–c).

To further investigate whether the peptide–TβRII complexes could facilitate the recruitment of TβRI, homology modeling was performed using the crystal structures of the TβRI–TβRII complex (PDB: 2L5S and 1PLO) and the TGF-β receptor complex (PDB: 2PJY). Based on these templates, two hypothetical trimeric models were constructed: TB1-TβRII-TβRI and TB2-TβRII-TβRI (Figure 2d–f).

### 2.2. Stability of Molecular Dynamics Simulations

For each of the four systems (TB1-TβRII, TB2-TβRII, TB1-TβRII-TβRI, and TB2-TβRII-TβRI), three independent replicates of unrestrained production molecular dynamics simulations were performed. After analysis, the results showed good consistency, and a representative trajectory from each system was selected for subsequent analysis. The stability of the four MD trajectories was analyzed in terms of root mean square deviation (RMSD) [44,45,46], root mean square fluctuation (RMSF) [47], radius of gyration (Rg) [48] and the number of hydrogen bonds.

#### 2.2.1. RMSD Analysis

Root mean square deviation (RMSD) quantifies the average displacement between corresponding atoms of two structures, typically using backbone atoms to evaluate conformational alignment and assess the stability of an MD system over time. In the present study, RMSD analysis was applied to assess the equilibrium states and structural stability of the peptide–receptor complexes across the 200 ns trajectories. The results demonstrated that the simulation time was sufficient to capture the dynamic properties of all four systems, each of which eventually reached global equilibrium.

**Figure 3:**
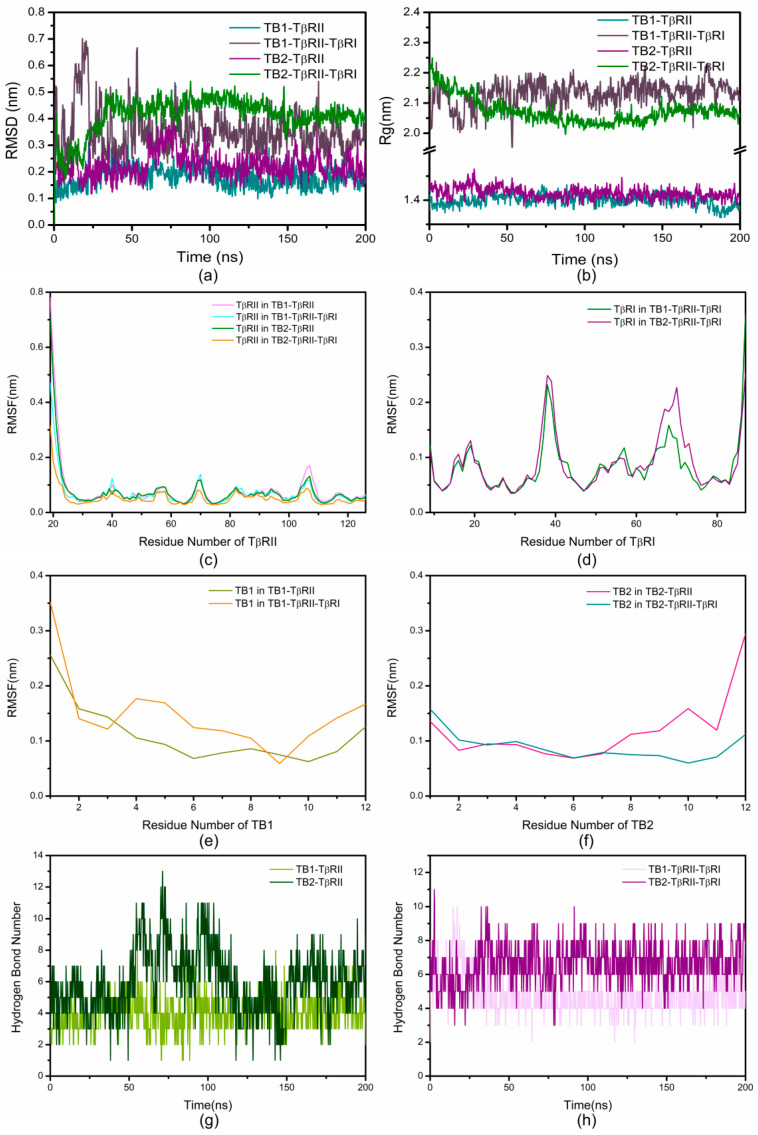
Trajectory Analysis of Molecular Dynamics Simulations. (**a**) RMSD analysis of TB1–TβRII (dark cyan curve), TB1–TβRII–TβRI (taro purple curve), TB2–TβRII (purple curve) and TB2–TβRII–TβRI (olive curve). (**b**) Rg analysis of TB1–TβRII (dark cyan curve), TB1–TβRII–TβRI (taro purple curve), TB2–TβRII (purple curve) and TB2–TβRII–TβRI (olive curve). (**c**) RMSF curves of TβRII calculated from the 200 ns trajectories of all four systems: TB1–TβRII (light magenta), TB1–TβRII–TβRI (light cyan), TB2–TβRII (olive), and TB2–TβRII–TβRI (orange). TβRII exhibited the largest fluctuation in the TB1–TβRII system and the smallest fluctuation in the TB2–TβRII–TβRI system. (**d**) RMSF of TβRI in the two trimeric systems: TB1–TβRII–TβRI (olive) and TB2–TβRII–TβRI (purple). (**e**) RMSF of TB1 in TB1–TβRII (dark yellow) and TB1–TβRII–TβRI (orange). (**f**) RMSF of TB2 in TB2–TβRII (pink) and TB2–TβRII–TβRI (dark cyan). (**g**) Hydrogen bond counts in the dimeric MD systems: TB1–TβRII (apple green, average = 4) and TB2–TβRII (dark green, average = 6). (**h**) Hydrogen bond counts in the trimeric MD systems: TB1–TβRII–TβRI (lilac, average = 4.7), with TβRI as the ligand and TB1–TβRII as the receptor; TB2–TβRII–TβRI (purple, average = 6.6), with TβRI as the ligand and TB2–TβRII as the receptor.

As shown in Figure 3a, TB1-TβRII and TB2-TβRII equilibrated after approximately 70 ns. The average RMSD values were 0.03376 nm for TB1-TβRII and 0.03654 nm for TB2-TβRII. In contrast, TB1-TβRII-TβRI reached global equilibrium only after ~100 ns and exhibited larger fluctuations, with an average RMSD of 0.05011 nm. These results suggest that although the trimeric TB1-TβRII-TβRI system ultimately achieved equilibrium, its conformation continued to undergo substantial dynamic transitions. By comparison, TB2-TβRII-TβRI equilibrated much earlier, at ~40 ns, with minimal fluctuations. The average RMSD was 0.0359 nm, indicating a more stable structural configuration during the simulation.

#### 2.2.2. Rg Analysis

The radius of gyration (Rg) reflects the overall compactness of a macromolecule and is defined as the root mean square distance of atoms from their common center of mass. A smaller Rg value indicates greater structural compactness and stability. To minimize interference from side-chain fluctuations, Rg values were calculated using only the protein backbone atoms.

As shown in Figure 3b, the Rg values of the TB1–TβRII and TB2–TβRII complexes reached equilibrium at approximately 40 ns and subsequently remained stable around 1.4 nm, with fluctuations confined within ~0.01 nm. This stability is consistent with the RMSD trends observed for these two systems. In contrast, the Rg trajectories of the trimeric complexes TB1–TβRII–TβRI and TB2–TβRII–TβRI exhibited distinct behaviors. For TB1–TβRII–TβRI, equilibrium was achieved at around 70 ns, but the Rg values displayed relatively large fluctuations, consistent with the dynamic instability revealed by RMSD analysis.

In the TB2–TβRII–TβRI system, however, the Rg values showed a sharp decline during the initial 0–40 ns, decreasing from 2.25 nm to 2.05 nm. Beyond 40 ns, the system entered a stable equilibrium phase that persisted until 200 ns, with minimal fluctuations. This rapid reduction in Rg indicates that the trimeric complex became more compact within the first 40 ns. These simulation results suggest that the specific binding pattern of TB2 to TβRII enhances its cooperative interaction with TβRI, thereby reinforcing the overall compactness and stability of the system.

#### 2.2.3. RMSF Analysis

Root mean square fluctuation (RMSF) is a key metric for assessing structural flexibility, as it quantifies the average deviation of each atom from its mean position over the course of the simulation. To evaluate residue-specific flexibility and positional variations, RMSF values of the Cα atoms were calculated for all four complexes. For consistency, the analysis was performed using the relatively stable equilibrium phase (100–200 ns) of each trajectory.

As shown in Figure 3c–f, the RMSF profiles of TβRII across the four complexes exhibited a high degree of consistency. The primary flexible region was located at the N-terminus (Ala19–Gln26), which constitutes the interaction interface with TβRI. The flexibility observed in this region may reflect lower predicted stability at the modeled interface of TβRI. Additional flexible segments of TβRII were identified at Thr37–Ser43, Glu55–Gln58, Asn68–Ile72, and Lys104–Gly107. Notably, the RMSF values of TβRII in the TB2–TβRII–TβRI system (orange curve in Figure 3c) were lower than in the other three systems, suggesting that the combined presence of TB2 and TβRI exerts a stabilizing effect.

For TβRI, the RMSF analysis of the two trimeric complexes revealed four major flexible regions: Cys14–Phe22, Ser33–Ser45, Pro55–Thr74, and the C-terminal residues Lys84–Leu87 (Figure 3d).

Figure 3e,f illustrate the RMSF values of TB1 and TB2 in both dimeric and trimeric complexes. For both peptides, the N- and C-terminal residues displayed the highest flexibility, a feature commonly observed for short peptides. The RMSF values of TB1 were higher in the trimeric complex compared to the dimeric system, suggesting that incorporation into the trimer did not restrict TB1’s conformational mobility. This observation may be attributed to the absence of direct interactions between TB1 and TβRI (Figure 3e).

Remarkably, it was observed that in the TB2–TβRII complex, TB2 exhibited reduced flexibility at its N-terminus but pronounced fluctuations at the C-terminus, with the RMSF of the terminal residue reaching 0.2943 nm (Figure 3f). This result implies that the binding of TB2 to TβRII is primarily mediated by its N-terminal region, whereas the C-terminus does not engage in stable interactions, which may also be the reason for the lower ipTM score of the AlphaFold3 TB2–TβRII model. Most notably, in the TB2–TβRII–TβRI system, the RMSF values of the TB2 C-terminus were markedly reduced compared to those in the dimeric complex, with the terminal residue showing an RMSF of only 0.1117 nm. This reduction indicates that the C-terminal region of TB2, due to the presence of TβRI, has its flexibility constrained, and it is likely that the C-terminal region of TB2 may form stabilizing interactions with TβRI.

These findings suggest that although both TB1 and TB2 act as binding peptides for TβRII, their dynamic behaviors differ significantly. The contrasting flexibilities and interaction patterns of TB1 and TB2 may help explain the observed differences, although experimental confirmation is required.

#### 2.2.4. Number of Hydrogen Bonds

The number of hydrogen bonds is a critical parameter for evaluating the strength and stability of intermolecular interactions, with a higher number of hydrogen bonds generally indicating stronger binding affinity. We first analyzed the hydrogen bonds formed between the peptides and TβRII, where the peptides were defined as ligands and TβRII as the receptor. The average number of hydrogen bonds between TB1 and TβRII was 4, whereas TB2 formed an average of 6 hydrogen bonds with TβRII (Figure 3g). These results suggest that TB2 may establish a more stable interaction with TβRII compared with TB1.

In the trimeric systems TB1–TβRII–TβRI and TB2–TβRII–TβRI, hydrogen bond analysis was performed with TβRI considered as the ligand and the TB–TβRII complexes as receptors. The average number of hydrogen bonds formed between TB1–TβRII and TβRI was 4.7, while the TB2–TβRII–TβRI system exhibited a higher value of 6.6 (Figure 3h). The greater number of hydrogen bonds observed for TB2 indicates stronger interactions and enhanced stability of the TB2–TβRII–TβRI complex compared with the TB1 counterpart.

### 2.3. Inter-Subunit Distances

The above analyses preliminarily suggested that in the TB2–TβRII–TβRI trimeric complex, TβRI interacts with the TB2–TβRII complex, whereas in the TB1–TβRII–TβRI trimer, such interactions appear absent. To further validate these observations, we analyzed the time-dependent distance changes between different subunits in all four systems.

**Figure 4:**
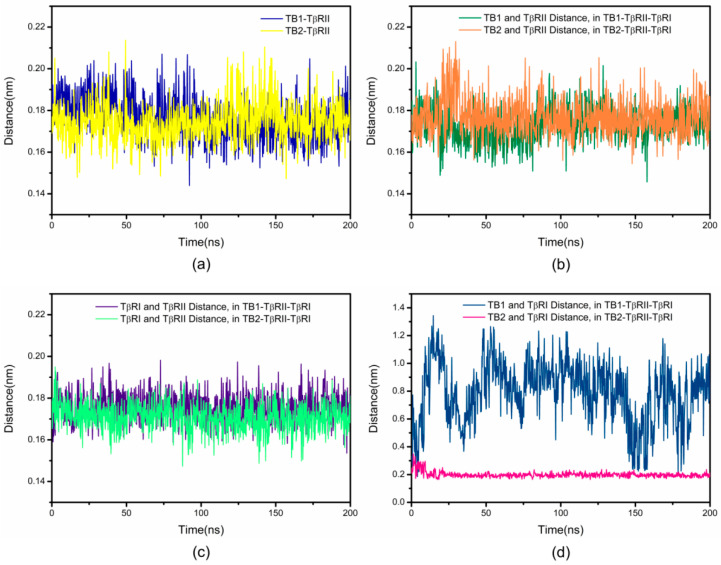
Inter-subunit distance analysis over the course of MD simulations. (**a**) Distance variations between TB1/TB2 and TβRII in the two dimeric systems. (**b**) Distance variations between TB1/TB2 and TβRII in the two trimeric systems. (**c**) Distance variations between TβRI and TβRII in TB1–TβRII–TβRI trimer (average = 0.175 nm) and TB2–TβRII–TβRI trimer (average = 0.170 nm). (**d**) Distance variations between TB1/TB2 and TβRI in TB1–TβRII–TβRI trimer (average = 0.790 nm) and TB2–TβRII–TβRI trimer (average = 0.199 nm).

As shown in Figure 4a, the distances between TBs and TβRII in the two dimeric complexes remained stable throughout the simulations. Similarly, in the trimeric systems (Figure 4b), the distances between TBs and TβRII were also stable. Analysis of the distances between TβRII and TβRI in the two trimeric models suggested relatively stable trends as well (Figure 4c). The average inter-receptor distance in the TB1 system was 0.175 nm, while in the TB2 system it was slightly shorter at 0.170 nm.

However, pronounced differences were observed in the distances between the peptides and TβRI (Figure 4d). In the TB2 trimer, the TB2–TβRI distance rapidly decreased from ~0.35 nm to ~0.19 nm within the first 20 ns and subsequently remained stable, with a fluctuation range of only 0.012 nm. In contrast, the TB1–TβRI distance exhibited large variations over time, without reaching a stable state, with an average distance of 0.790 nm and an average fluctuation of 0.1990 nm.

Taken together, these results indicate that in the TB2–TβRII–TβRI trimer, TB2 establishes a clear and stable interaction with TβRI, rapidly approaching and maintaining close contact. In contrast, TB1 shows little to no association with TβRI, consistent with the absence of stable interactions in the TB1–TβRII–TβRI system.

### 2.4. Free Energy Landscape (FEL)

Free energy landscape (FEL) analysis based on principal component analysis (PCA) is a powerful approach for characterizing the energetic stability and conformational states of protein–ligand complexes [49]. PCA was performed for the four systems—TB1–TβRII, TB2–TβRII, TB1–TβRII–TβRI, and TB2–TβRII–TβRI—using the first two principal components (PC1 and PC2) to calculate the Gibbs free energy of each conformation and construct three-dimensional free energy landscapes (3D FELs). PCA captures the dominant motions of proteins during simulations, while the FELs, projected onto the PC1–PC2 space, map Gibbs free energy variations through surface height and color gradients, reflecting transitions from high-energy to low-energy states.

**Figure 5:**
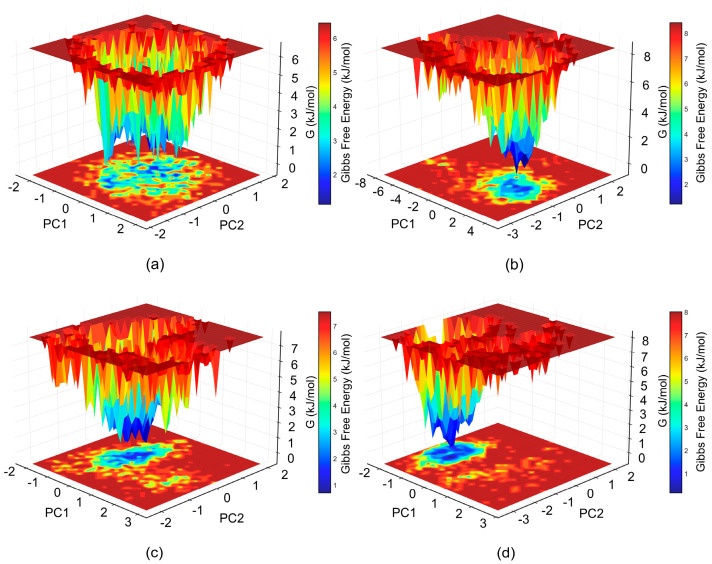
Principal component-based free energy landscape (FEL) analysis. (**a**) FEL of the TB1–TβRII complex. (**b**) FEL of the TB1–TβRII–TβRI complex. (**c**) FEL of the TB2–TβRII complex. (**d**) FEL of the TB2–TβRII–TβRI complex. The color gradient from blue to red represents the Gibbs free energy from low to high. The horizontal plane corresponds to the first (PC1) and second (PC2) principal components. The well-defined energy minimum of the TB2 trimer correlates with its restricted conformational dynamics and high functional stability.

As shown in Figure 5, deeper blue regions correspond to low-energy conformations, representing the most stable states. The TB1–TβRII complex exhibited multiple energy basins (Figure 5a), suggesting that this system explored several conformational substates during the simulation, indicative of conformational diversity. In contrast, TB1–TβRII–TβRI (Figure 5b), TB2–TβRII (Figure 5c), and TB2–TβRII–TβRI (Figure 5d) each displayed well-defined energy minima, consistent with strong thermodynamic stability. Collectively, the FEL analysis strongly supports our findings that TB2, unlike TB1, forms stable conformations upon binding to TβRII. Furthermore, the TB2–TβRII–TβRI complex maintains a stable conformation throughout the simulations, supporting the computational hypothesis of TB2-mediated receptor engagement.

### 2.5. TB2 Peptide Movement

**Figure 6:**
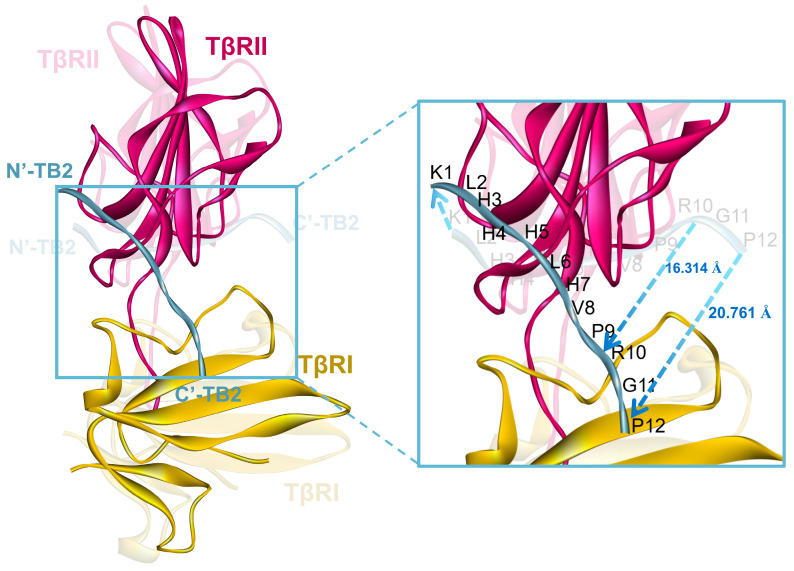
TB2 Peptide Movement. The initial conformation (shown in semi-transparent representation at the back) is superimposed with the representative conformation (front). R1 and R2 are highlighted in yellow and purple, respectively, and the TB2 peptide is shown in gray. The peptide movement of TB2 from the initial to the representative conformation is indicated by blue arrows. Notably, the C-terminal residues of TB2 undergo significant displacement.

To further elucidate the direction of movement of TB2 within the TB2–TβRII–TβRI system and clarify whether it facilitates the recruitment of TβRI, we extracted the most energetically favorable representative conformation of the TB2–TβRII–TβRI system from the energy basin identified in the FEL analysis. Structural superposition of the initial conformation with this representative conformation was then performed to analyze the direction of TB2 peptide movement. As shown in Figure 6, during the dynamic transition of TB2–TβRII–TβRI, the TB2 peptide underwent a significant positional shift. This movement was particularly pronounced in the C-terminal residues, with Pro9, Arg10, Gly11, and Pro12 all exhibiting displacements exceeding 10 Å. Most notably, the Cα atom of Pro12 displayed a displacement of 20.761 Å (Table 1). Upon binding to TβRII, the N-terminus of TB2 exhibited a certain degree of movement but remained associated with TβRII. In contrast, the C-terminus underwent a substantial shift toward TβRI, consistent with the results obtained from RMSF, Rg, and distance analyses. In addition to TB2, conformational adjustments were also observed in both TβRI and TβRII.

Taken together, these findings further substantiate our earlier conclusions: TB2 may behave similarly to TGF-β3 in terms of predicted receptor engagement. Specifically, after the N-terminus of TB2 binds to TβRII, its C-terminus progressively and rapidly approaches TβRI, ultimately anchoring to TβRI and facilitating the formation of a stable TB2–TβRII–TβRI trimeric complex. This structural recruitment of TβRI by TB2 is likely not only a structural phenomenon but may also be reflected in the interaction network and energy landscape of the system.

### 2.6. Dynamic Cross-Correlation Matrix (DCCM)

The Dynamic Cross-Correlation Matrix (DCCM) provides insights into the coordinated motions of residues within a protein complex [50]. To further elucidate residue-specific correlated movements and internal dynamics of the TB2–TβRII–TβRI complex, we calculated the covariance matrix based on the trajectories of Cα atoms and generated the corresponding DCCM.

**Figure 7:**
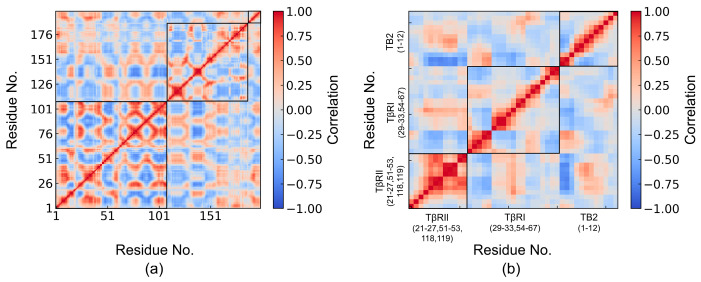
Dynamic cross-correlation matrix (DCCM) analysis of the TB2–TβRII–TβRI complex. (**a**) DCCM analysis of the entire system, including all 199 residues spanning the three subunits in sequence: TβRII, TβRI, and TB2. (**b**) DCCM analysis of the inter-subunit interaction regions, defined as all Cα atoms of residues within 5 Å of any contact surface between subunits. The color gradient from deep blue to deep red represents the correlation values from fully anti-correlated to fully correlated, respectively.

As shown in Figure 7, DCCM values range from +1 to −1, where +1 (deep red) represents fully correlated motions and –1 (deep blue) represents fully anti-correlated motions. The intensity of the color indicates the degree of correlation, with darker colors reflecting stronger associations. Initially, DCCM analysis was performed for the entire TB2–TβRII–TβRI system, comprising 199 residues (Figure 7a).

To focus on the interfacial interactions, we defined a specific subgroup consisting of residues within 5 Å of inter-subunit atomic contacts (Cα atoms only): TβRII (residues 21–27, 51–53, 118–119), TβRI (residues 29–33, 54–67), and TB2 (residues 1–12). The DCCM of this subgroup is shown in Figure 7b. Notably, TβRI contributed more residues (19 residues) to the interfacial interactions than TβRII (12 residues). Residue 58 of TβRI displayed dynamic correlations with multiple residues of TβRII during the MD simulation. TB2 residues 1–3 exhibited dynamic correlations with TβRI residues 30–33 as well as several residues of TβRII; residues 4–7 correlated with TβRII residues 51–53; residues 6–9 correlated with TβRII residues 24–27; and residues 10–12 correlated with TβRI residues 58–63.

These results suggest that nearly all residues of TB2 are actively involved in interactions with both TβRII and TβRI, suggesting a potential role in bridging the two receptors and stabilizing the trimeric complex.

### 2.7. Key Hydrogen Bond Interactions

Hydrogen bond formation plays a critical role in stabilizing protein structures and mediating protein recognition processes. In the TB2–TβRII–TβRI complex, we observed variations in hydrogen bond formation among the three subunits (Table 2). Using an occupancy threshold of ≥40% during MD simulations, a total of 12 hydrogen bonds were identified between the subunits.

Notably, a highly stable hydrogen bond was formed between the hydrogen atom of Gly11 in TB2 and the oxygen atom of Phe60 in TβRI, exhibiting an occupancy of 77% and a distance of 0.293 ± 0.014 nm. This interaction is crucial for stabilizing the TB2–TβRI interface.

TB2 also established multiple hydrogen bonds with TβRII, among which several were particularly significant: (1) the nitrogen atom of His5 in TB2 formed a stable hydrogen bond with the hydrogen atom of Thr51 in TβRII (occupancy = 92.9%, distance = 0.307 ± 0.014 nm); (2) the hydrogen atom of Leu6 in TB2 formed a hydrogen bond with the oxygen atom of Thr51 in TβRII (occupancy = 88.4%, distance = 0.302 ± 0.017 nm); and (3) the oxygen atom of Glu119 in TβRII formed three hydrogen bonds with the hydrogen atoms of Arg10 in TB2, with occupancies of 49.1%, 45.5%, and 44.3%, respectively.

Similarly, multiple stable hydrogen bonds were observed between TβRI and TβRII, with four exhibiting occupancies greater than 90%: (1) the hydrogen atoms of Arg58 in TβRI formed two hydrogen bonds with the oxygen atom of Asp118 in TβRII, showing occupancies of 98.6% (distance = 0.277 ± 0.010 nm) and 97.0% (distance = 0.282 ± 0.011 nm); (2) the hydrogen atom of Arg58 in TβRI formed a hydrogen bond with the oxygen atom of Pro25 in TβRII (occupancy = 95.9%, distance = 0.285 ± 0.011 nm); and (3) the hydrogen atom of Cys76 in TβRI formed a hydrogen bond with the oxygen atom of Val22 in TβRII (occupancy = 91.2%, distance = 0.298 ± 0.015 nm).

These hydrogen bond interactions may collectively contribute to the stabilization of the TB2–TβRII–TβRI trimeric complex and likely underlie the high binding affinity and functional cooperation observed in this system.

### 2.8. Salt Bridge Interactions

In protein complexes, salt bridges are formed between oppositely charged amino acid residues when the distance between their side-chain centroids falls within a defined threshold and the geometric angle satisfies specific criteria. As an important stabilizing force for protein tertiary structures and molecular recognition, salt bridges are crucial for understanding biomolecular interactions. A salt bridge is considered stable when the distance between the charged side-chain centroids remains consistently within 3.5–5.0 Å. The dynamic behavior and stability of these salt bridges directly influence the overall stability of the complex.

**Figure 8:**
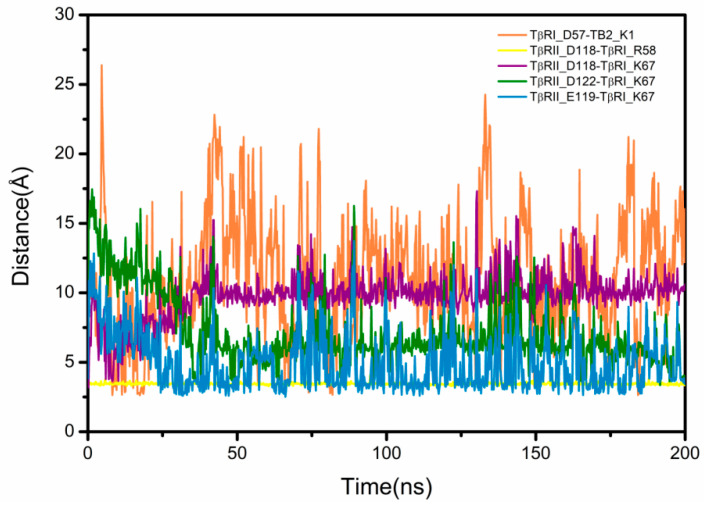
Salt bridge analysis of the TB2–TβRII–TβRI complex. Five salt bridges were identified: TβRI Asp57–TB2 Lys1, TβRII Asp118–TβRI Arg58, TβRII Asp118–TβRI Lys67, TβRII Asp122–TβRI Lys67, and TβRII Glu119–TβRI Lys67. Using the distance analysis tool in GROMACS, the temporal variations of the Cα–Cα distances for these salt bridges during MD simulation are shown as yellow, blue, green, purple, and orange curves, respectively.

Using the salt bridge analysis tool in VMD [51], we examined the TB2–TβRII–TβRI complex and identified several salt bridge interactions during the interaction between TβRI and the TB2–TβRII complex (Figure 8). Key salt bridges included TβRI_Asp57–TB2_Lys1, TβRII_Asp118–TβRI_Arg58, TβRII_Asp118–TβRI_Lys67, TβRII_Asp122–TβRI_Lys67, and TβRII_Glu119–TβRI_Lys67. We further analyzed the temporal variation of the Cα–Cα distances for these salt bridges to evaluate their stability over the simulation.

The salt bridge between TβRII_Asp118 and TβRI_Arg58 (yellow) maintained an extremely close distance throughout the simulation, with over 95% of the time below 4 Å, consistent with the characteristics of a strong salt bridge. Additionally, the TβRII_Glu119–TβRI_Lys67 interaction (blue) also remained relatively stable, primarily fluctuating within 2–8 Å, although transient dissociation events were observed. The dynamic variation in these salt bridge distances indicates the presence of persistent ionic interactions between TβRI and TβRII, which may significantly enhance inter-subunit binding through charge complementarity. The other three salt bridge pairs mostly remained above 4 Å during the MD simulations and did not exhibit pronounced salt bridge characteristics.

### 2.9. Relative Binding Free Energy Calculation

To investigate the relative binding affinities of TB peptides to TβRII and their potential to recruit TβRI, we performed MM-PBSA [52,53] calculations on the four systems. As shown in Table 3, TB2–TβRII exhibited a relative binding free energy of −35.91 ± 9.29 kcal/mol, while TB1–TβRII showed −20.35 ± 7.89 kcal/mol, indicating that both peptides can bind TβRII, with TB2 displaying higher affinity, consistent with previous experimental observations. For the trimeric complexes, the binding free energies between TβRI and the TB–TβRII complexes were −67.76 ± 7.70 kcal/mol for TB2–TβRII–TβRI and −38.12 ± 4.07 kcal/mol for TB1–TβRII–TβRI. Decomposition of the binding energy revealed that in TB2–TβRII–TβRI, TβRI interacts with TβRII (ΔG_binding = −47.19 ± 5.94 kcal/mol) and TB2 (ΔG_binding = −19.58 ± 4.00 kcal/mol), whereas in TB1–TβRII–TβRI, TβRI exhibits negligible binding to TB1 (ΔG_binding = −0.18 ± 0.11 kcal/mol) and primarily binds TβRII (ΔG_binding = −37.94 ± 4.06 kcal/mol).

Further analysis of energy components showed that van der Waals interactions (ΔG_vdw) and electrostatic interactions (ΔG_ele) are the major contributors to the stability and affinity of TB2–TβRII–TβRI, with ΔG_vdw = −76.12 ± 5.09 kcal/mol and ΔG_ele = −205.01 ± 34.61 kcal/mol, compared to ΔG_vdw = −37.05 ± 5.79 kcal/mol and ΔG_ele = −84.78 ± 21.33 kcal/mol in TB1–TβRII–TβRI. Although the polar solvation energy partially opposes binding, strong van der Waals and electrostatic interactions compensate for this unfavorable contribution, whereas the effect of nonpolar energy is minor.

Taken together, the computational results indicate that formation of the TB2–TβRII complex not only stabilizes the peptide–receptor interaction but also actively recruits and stably binds TβRI, with all three components contributing positively to complex formation. In contrast, TB1 binding to TβRII does not promote TβRI recruitment, highlighting the differential biological activities of the two peptides. To reduce computational costs, the MM-PBSA calculations in this study omitted entropic contributions. This represents a notable limitation, particularly for flexible peptide systems, as neglecting entropy may overestimate receptor–ligand binding affinities. Consequently, the resulting relative binding free energies are suitable only for comparative analysis of ligand binding within similar systems and should not be interpreted as absolute binding free energies.

### 2.10. Per-Residue Energy Decomposition

**Figure 9:**
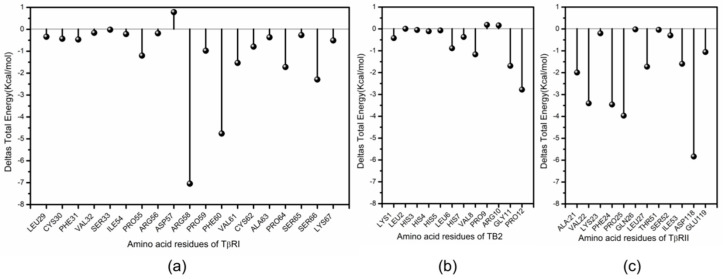
Residue-wise energy contribution analysis of key amino acids in the TB2–TβRII–TβRI complex. (**a**) Energy contributions of key residues in TβRI, including residues 29–33 and 54–67. (**b**) Energy contributions of key residues in TβRII, including residues 21–27, 51–53, and 118–119. (**c**) Energy contributions of amino acid residues in TB2.

To elucidate the contributions of individual amino acid residues to the relative binding free energy in the TB2–TβRII–TβRI complex, we performed per-residue energy decomposition at the interaction interface between TβRI and the TB2–TβRII complex, as well as for all residues in TB2. The analyzed residues include TβRI: 29–33, 54–67; TβRII: 21–27, 51–53, 118–119; TB2: 1–12 (Figure 9).

In TβRI, residues Cys30, Phe31, Pro55, Arg58, Pro59, Phe60–Pro64, Ser66, and Lys67 contributed significantly to binding, with per-residue energies below −0.3 kcal/mol. In TβRII, residues Ala21, Val22, Phe24, Pro25, Leu27, Ile53, Asp118, and Glu119 showed notable contributions (energy < −0.3 kcal/mol). In TB2, the primary energy-contributing residues were located at the C-terminal region of the peptide, especially Leu6, His7, Val8, Gly11, and Pro12.

Among these, TβRII Asp118 (−5.833 kcal/mol), TβRI Arg58 (−7.045 kcal/mol) and Phe60 (−4.756 kcal/mol), as well as TB2 Gly11 (−1.69 kcal/mol) and Pro12 (−2.779 kcal/mol), play crucial roles in facilitating the recruitment of TβRI by the TB2–TβRII complex. Conversely, some residues at the interface, such as TβRI Asp57 and TB2 Pro9 and Arg10, exhibited positive energy contributions (>0 kcal/mol), suggesting a minor inhibitory effect on the binding of TB2–TβRII to TβRI, although this effect is limited.

By integrating hydrogen bond, salt bridge, and per-residue energy decomposition analyses, we identified key residues that facilitate the recruitment of TβRI by the TB2–TβRII complex (Table 4). In TβRI, Arg58 forms three stable hydrogen bonds with TβRII Asp118 and Pro25 (occupancies 98.6%, 97%, and 95.9%) and a salt bridge with Asp118, contributing strongly via electrostatic (−46.333 kcal/mol) and van der Waals (−4.67 kcal/mol) interactions. Phe60 forms a hydrogen bond with TB2 Gly11 (occupancy 77.8%) and contributes −5.804 kcal/mol via van der Waals interactions, while Ser66 hydrogen bonds with TβRII Asp118 (occupancy 46.7%) with an electrostatic contribution of −8.091 kcal/mol. In TβRII, Asp118 and Glu119 form multiple hydrogen bonds with TB2 and TβRI, contributing significantly through electrostatic interactions (−35.103 and −27.26 kcal/mol, respectively), and Val22 and Pro25 also form stabilizing hydrogen bonds with TβRI. On the TB2 peptide, Gly11 forms a critical hydrogen bond with Phe60, contributing −1.69 kcal/mol overall, and Pro12 contributes mainly via polar solvation and van der Waals interactions. Collectively, these residues constitute key interaction hotspots that stabilize the TB2–TβRII–TβRI complex and enable efficient recruitment of TβRI.

### 2.11. Experimental Validation of Peptide Activation of the Smad Pathway

**Figure 10:**
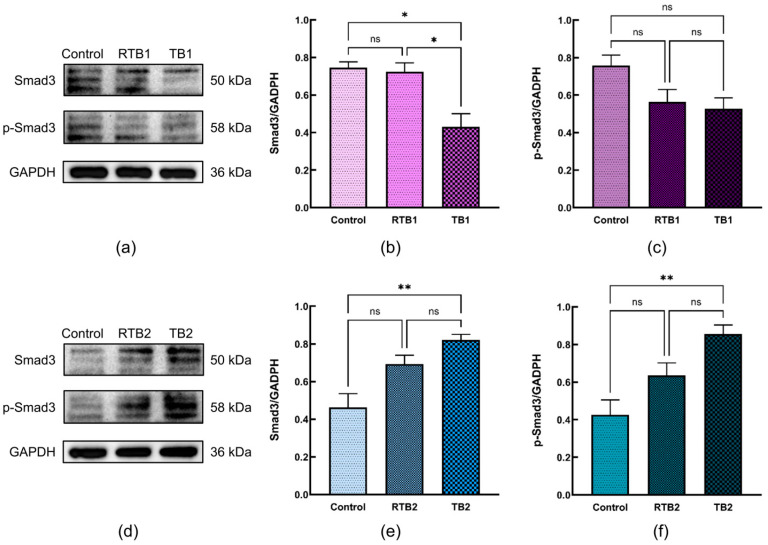
The erffects of TB1, TB2, and their respective scrambled control peptides on Smad3 and p-Smad3 expression in 3T3 cells. (**a**) Western blot analysis of TB1 and RTB1. GAPDH (glyceraldehyde-3 phosphate dehydrogenase) served as the loading control. RTB1 is a scrambled peptide with the same amino acid composition as TB1. TB1 treatment markedly reduced Smad3 levels compared to the blank control, whereas RTB1 showed no significant effect. (**b**) Quantification of Smad3 normalized to GAPDH. Smad3 expression in the TB1 group was significantly lower than in the control (* *p* < 0.05) and RTB1 group (*p* < 0.05). (**c**) Quantification of p-Smad3/GAPDH. TB1 treatment slightly reduced p-Smad3 levels compared to control and RTB1, but the differences were not statistically significant. (**d**) Western blot analysis of TB2 and RTB2. GAPDH was used as the loading control. TB2 treatment significantly increased Smad3 levels compared to both control and RTB2. (**e**) Quantification of Smad3/GAPDH. Smad3 expression in the TB2 group was significantly higher than control (** *p* < 0.01). (**f**) Quantification of p-Smad3/GAPDH. TB2 treatment significantly elevated p-Smad3 levels relative to control (** *p* < 0.01).

Western blot (WB) analysis was performed to experimentally validate the peptide effects on the TGF-β/Smad signaling pathway and corroborate molecular simulation predictions. It is important to note that this method confirms the functional divergence between TB1 and TB2, rather than directly assessing receptor binding or recruitment events. TB1, TB2, and their corresponding random-sequence controls (RTB1: RPMIANQGDSHL; RTB2: VLGHKHPHRHLP) were applied to 3T3 cells for 48 h, with untreated cells serving as the control. As shown in Figure 10a–c, TB1 treatment significantly reduced Smad3 protein expression compared with both the control and RTB1 groups, while the level of phosphorylated Smad3 (p-Smad3) decreased, but the change did not reach statistical significance. These results indicate that TB1 suppresses Smad3 expression, reflecting an inhibitory effect on the TGF-β/Smad signaling pathway. This observation aligns with our computational analyses, which suggest that although TB1 binds the extracellular domain of TβRII, its binding site may hinder TβRI recruitment by TβRII.

In contrast, TB2 treatment markedly increased both Smad3 and p-Smad3 protein levels relative to the control and RTB2-treated groups (Figure 10d–f), confirming that TB2 effectively activates the TGF-β/Smad signaling pathway. The random-sequence control peptide RTB2 did not produce similar effects, emphasizing the sequence-specific activity of TB2. These experimental findings are in line with our molecular dynamics (MD), principal component analysis (PCA), dynamic cross-correlation matrix (DCCM), and MM-PBSA analyses, which suggest that TB2 not only forms a stable complex with TβRII but also facilitates TβRI recruitment, thereby enhancing Smad3 expression and triggering downstream signaling. This mechanistic insight likely underlies previous observations that TB2 promotes the secretion of type I and type III collagen.

## 3. Discussion

In this study, we investigated the biological functional differences between two TGF-β-mimetic peptides targeting TβRII, TB1 and TB2, through a multidimensional approach encompassing structural analysis, dynamic behavior, and experimental validation. The main findings were as follows: (1) TB2 forms a complex with TβRII via its N-terminal region, and its binding domains (Leu27–Asp32 and Ser49–Glu55) highly overlap with the TGF-β binding sites on TβRII. (2) MD simulations predict that the TB2–TβRII complex further recruits TβRI to form a stable heterotrimer, with a calculated relative binding free energy of −67.76 ± 7.70 kcal/mol. (3) Key interaction sites include the C-terminal residues of TB2 (Arg10, Gly11 and Pro12) and the N-terminal flexible region of TβRII (Ala19–Leu26). Notably, a stable hydrogen bond is formed between the H atom of TB2 Gly11 and the O atom of TβRI Phe60 (occupancy: 77.8%), and between TβRI Arg58 and TβRII Asp118 (occupancy: 97%). Additionally, a stable salt bridge is observed between TβRII Glu119 and TβRI Lys67. (4) Western blot experiments confirmed that TB2 activates the Smad3-dependent TGF-β/Smad signaling pathway.

Taken together, our computational modeling suggests that TB2 is predicted to mimic TGF-β by binding to TβRII and recruiting TβRI, thereby stabilizing the trimeric receptor complex, activating the Smad-dependent TGF-β/Smad signaling pathway, and promoting collagen secretion. In contrast, TB1 binds TβRII but appears unable to recruit TβRI, resulting in ineffective pathway activation. These findings not only validate the computational predictions but also suggest TB2 as a promising peptide template meriting further experimental evaluation for the rational design of therapeutics targeting the TGF-β/Smad signaling pathway and tissue repair. Of course, all results and conclusions remain subject to future experimental validation, such as through receptor-binding assays (e.g., SPR or ITC) and structural characterization techniques, to confirm the predicted molecular interactions.

In summary, by integrating AlphaFold 3 modeling, molecular dynamics (MD) simulations, relative binding free energy calculations, and experimental validation, this study proposes a possible molecular mechanism by which the peptide TB2 mimics TGF-β function and activates the TGF-β/Smad signaling pathway. The approaches and findings presented herein provide a valuable reference for the development of novel therapeutics for tissue repair.

## 4. Materials and Methods

### 4.1. Modeling of TB1–TβRII and TB2–TβRII Using AlphaFold3

AlphaFold3 [33,34,35], developed by DeepMind (Google DeepMind, 2024, London, UK), represents the latest generation of AI-based models for biomolecular structure prediction, leveraging deep learning algorithms to achieve high-accuracy structural forecasts. Initial structure predictions were performed using the AlphaFold3 online server (https://alphafoldserver.com/) with the default system parameters, which have been extensively optimized based on experimental data to balance computational efficiency and predictive accuracy in most scenarios.

For template searching, the default parameters were applied to ensure broad and effective homology template selection. The number of iterative predictions was set to 20, a parameter widely validated in previous studies to provide reliable structural predictions while maintaining computational efficiency. In addition, internal random seeds were used to enable reproducibility and account for stochastic variation in model predictions. The Pairformer module of AlphaFold3 was employed to process sequence alignments and generate the initial three-dimensional structures of the complexes. Following multi-step optimization of atomic coordinates and iterative refinement, final complex models were obtained and visualized using Discovery Studio v4.5 (DS45) [54]. A comprehensive evaluation strategy combining energy-based scoring and structural similarity comparison was applied.

The quality of the predicted structure was evaluated using the interface predicted Template Modeling score (ipTM) and the predicted Template Modeling score (pTM) to assess the accuracy of the interaction interfaces between different subunits (chains), as well as the Expected Position Error (EPE) of overall three-dimensional folding structure of the entire complex.

### 4.2. Construction of TB1–TβRII–TβRI and TB2–TβRII–TβRI Hypothetical Models

Based on the hypothesis proposed in this study, the TGF-β mimetic peptides are expected to simulate the biological function of TGF-β by binding to TβRII and further enhancing the affinity between TβRII and TβRI. Accordingly, we constructed the hypothetical models of TB1–TβRII–TβRI and TB2–TβRII–TβRI by incorporating the TβRI structural region into the previously obtained TB1–TβRII and TB2–TβRII complexes.

Model construction was performed using the Homology Modeling [55,56,57] module in Discovery Studio v4.5 (DS45) [54]. The crystal structures of the TGF-β–receptor complex (PDB ID: 2PJY, containing a TβRI–TβRII–TGF-β3 trimer) [16] and the individual receptors TβRI (PDB ID: 2L5S) [58] and TβRII (PDB ID: 1PLO) [59] were employed as templates. The Align Structures function in DS45 was used to spatially superimpose the AF3-generated TB1–TβRII and TB2–TβRII complexes onto the templates. This alignment is based on multiple sequence alignment (MSA), with a gap opening penalty set to 3.0.

Subsequently, the DS_Modeler module [56] was used to build the initial structures of the TB1–TβRII–TβRI and TB2–TβRII–TβRI models. Energy minimization was then performed in the GBSW implicit solvent model using the native DS protein force field via the DS_CHARMM [60] module, with a convergence criterion of 0.4184 kJ·mol^−1^·nm^−1^. These minimized structures were used as starting points for the subsequent molecular dynamics simulations.

### 4.3. Molecular Dynamics (MD) Simulations

Molecular dynamics (MD) simulations provide a powerful approach for modeling protein conformational changes under physiological conditions, thereby offering critical insights into the dynamic interactions between ligands and receptors [37,39]. To investigate the dynamic properties of TB1–TβRII, TB2–TβRII, TB1–TβRII–TβRI, and TB2–TβRII–TβRI, we performed 200 ns long-term molecular dynamics (MD) simulations using the GROMACS (Version 2024.2) package [61,62] with the RSFF2C forcefield [63,64] to examine atomic-level interactions and dynamic energetic behavior. Each initial model was solvated in a cubic box containing TIP3P [65] water molecules, and physiological ion conditions were established by adding Na^+^ and Cl^−^ ions to a concentration of 0.15 M, ensuring overall charge neutrality. Then, the protein molecules were subjected to 1000 steps of steepest descent (SD) energy minimization to remove steric clashes. Subsequently, a 2 ns restrained MD simulation of water molecules and ions was performed (1 ns each in the NVT and NPT ensembles) [66,67,68]. Finally, three independent replicates of unrestrained production MD simulations were conducted for 200 ns under the NPT ensemble at 300 K and 1 atmospheric pressure (atm) with periodic boundary conditions (PBC) [69]. Electrostatic interactions were calculated using the Particle-Mesh Ewald (PME) method, all the hydrogen bond lengths were constrained with the LINCS algorithm [70], the integration time step was set to 2 fs, and configurations were recorded every 200 ps.

The resulting MD trajectories were analyzed for Root Mean Square Deviation (RMSD) [44,45,46], Root Mean Square Fluctuation (RMSF) [47], Radius of Gyration (Rg) [48], and the number of hydrogen bonds to assess system stability. Additionally, inter-subunit distance analysis and principal component analysis (PCA)-based free energy landscape (FEL) calculations [49] were performed to further characterize the dynamic behavior of TB1 and TB2 during the MD simulations.

### 4.4. Dynamical Cross-Correlation Matrix (DCCM) Analysis

The Dynamical Cross-Correlation Matrix (DCCM) analysis [50] is an essential approach for investigating coordinated motions in functional biomacromolecules, such as proteins. DCCM captures the correlated motion between individual residues within a protein, allowing identification of residues that exhibit similar dynamic behavior during molecular motions. In receptor–ligand systems, conformational changes in the receptor and ligand are often interdependent, exhibiting coordinated motion. DCCM quantifies these correlations, providing insights into how ligand binding affects protein dynamics and highlighting key residues at the interaction interface that contribute to binding, thereby elucidating the mechanistic basis of protein–ligand interactions. For a system comprising N atoms, the DCCM is calculated as follows:

(1) $$C_{ij}=\frac{\left\langle \Delta r_{i}\cdot \Delta r_{j}\right\rangle }{\sqrt{\left\langle \Delta r_{i}^{2}\rangle \langle \Delta r_{j}^{2}\right\rangle }}$$

Among them, the correlation coefficient C_ij_ ranges from −1 to 1, and Δr_i_ = r_i_(t) − <r_i_>, where r_i_(t) is the position vector of the atom at time t, and <r_i_> is the average of the atom’s position vector over the entire trajectory time range; the angle brackets <⋅> denote the average operation over the entire trajectory time. We used the covar tool in the GROMACS software (Version 2024.2) package to calculate the covariance of pairwise amino acid residues. In the representative conformations of the Free Energy Landscape (FEL), we selected the alpha carbon atoms of amino acid residues at the interaction interfaces between TB2 and TβRII, between TβRII and TβRI, and between the TB2 polypeptide and TβRI, to obtain the correlation of motion patterns between pairwise residues.

We utilized the GROMACS covar tool to compute the covariance matrix for paired amino acid residues. For the representative conformation extracted from the Free Energy Landscape (FEL) analysis, we selected Cα atoms at the following interaction interfaces: between TB2 and TβRII, between TβRII and TβRI, and between the TB2 peptide and TβRI. The motions of these paired residues were analyzed to quantify correlations in their dynamic behavior.

### 4.5. Relative Binding Free Energy Calculation by MM-PBSA

Relative binding free energy was calculated using the MM-PBSA (Molecular Mechanics Poisson–Boltzmann Surface Area) method, which combines molecular mechanics (MM) energies, polar solvation energy (PB), and nonpolar solvation energy (SA). PB and SA describe solvent effects, while MM energy accounts for van der Waals and electrostatic interactions between the receptor and ligand. A negative energy indicates spontaneous binding, with lower values corresponding to stronger binding affinity.

Fifty representative snapshots were extracted from the equilibrium phase of each MD trajectory for relative binding free energy calculation and per-residue energy decomposition using the gmx_MMPBSA (Version 1.63) software package [52,53]. In the MM-PBSA [40,41,42,71] approach, the enthalpic contribution of the system is evaluated using molecular mechanics (MM). The polar component of solvation free energy is calculated by solving the Poisson-Boltzmann (PB) equation, whereas the nonpolar contribution is estimated based on the solvent-accessible surface area (SA). The overall relative binding free energy can be expressed as follows:

(2) $$\begin{array}{ll} \Delta G_{bind} & =\Delta E_{MM}+\Delta G_{sol}-T\Delta S\\ & =\Delta E_{MM}+\Delta G_{PB}+\Delta G_{SA}-T\Delta S\\ & =\Delta E_{vdw}+\Delta E_{ele}+\Delta G_{PB}+\Delta G_{SA}-T\Delta S \end{array}$$

where ΔG_bind_ represents the relative binding free energy, ΔE_MM_ denotes the molecular mechanical energy difference in vacuum, comprising electrostatic interactions (ΔE_ele_) and van der Waals interactions (ΔE_vdw_); ΔG_sol_ corresponds to the solvation free energy difference, which includes the polar solvation contribution (ΔG_PB_), calculated by solving the finite-difference Poisson-Boltzmann equation, and the nonpolar solvation contribution (ΔG_SA_), estimated from the solvent-accessible surface area (SASA). T is the absolute temperature, ΔS is the entropy change, and to save computational costs and improve computational efficiency, the entropic term (TΔS) was omitted in MM-PBSA calculations.

### 4.6. Peptide Synthesis and Western Blot (WB) Analysis

The peptides TB1 and TB2 and their corresponding random-sequence controls, RTB1 and RTB2 (sequences synthesized by Sangon Biotech, Shanghai, China; purity > 95%), were dissolved in sterile PBS to prepare 1 mM stock solutions. To avoid artificial charge interactions, the peptides were synthesized with N-terminal acetylation and C-terminal amidation. Mouse embryonic fibroblasts (NIH/3T3 cells) were used as an in vitro model system. Cells were seeded in 6-well plates at a density of 5 × 10^5^ cells per well and cultured for 24 h prior to treatment. The experimental design included the following groups: 1. Control group: Cells maintained in complete culture medium without peptide treatment. 2. Treatment groups: Cells treated with TB1 (0.2 mg/mL), TB2 (0.2 mg/mL), RTB1 (0.2 mg/mL), or RTB2 (0.2 mg/mL) for 48 h. After treatment, cells were harvested by trypsinization and subjected to Western blotting to evaluate protein expression. GAPDH (glyceraldehyde-3 phosphate dehydrogenase) was used as the internal loading control. Primary antibodies against Smad3, phospho-Smad3 (p-Smad3), and GAPDH were applied according to the manufacturer’s instructions. Protein bands were quantified using ImageJ (Version v1.54r), and the relative protein expression levels were normalized to GAPDH. Statistical analysis was performed using one-way ANOVA in GraphPad Prism 9.0 (GraphPad Software Inc., Boston, MA, USA), with *p* < 0.05 considered statistically significant.

## Figures and Tables

**Table 1:** The displacements of the Cα atoms for the 12 amino acid residues of TB2.

Amino Acid Residues of TB2	Displacement (Å)
LYS1	4.971
LEU2	6.860
HIS3	6.057
HIS4	5.671
HIS5	1.369
LEU6	2.414
HIS7	4.050
VAL8	9.777
PRO9	12.394
ARG10	16.314
GLY11	19.459
PRO12	20.761

**Table 2:** Key hydrogen bond interactions.

Donor and Acceptor	Occupancy (%)	Distance (nm)
TB2_GLY11:H–TβRI_PHE60:O	77.8	0.293 ± 0.014
TβRII_THR51:H–TB2_HIS5:ND1	92.9	0.307 ± 0.014
TB2_LEU6:H–TβRII_THR51:O	88.4	0.302 ± 0.017
TB2_ARG10:HH22–TβRII_GLU119:OE1	49.1	0.282 ± 0.014
TβRII_SER52:HG–TB2_HIS4:O	46.1	0.280 ± 0.016
TB2_ARG10:HH12–TβRII_GLU119:OE2	45.5	0.291 ± 0.019
TB2_ARG10:HH12–TβRII_GLU119:OE1	44.3	0.292 ± 0.019
TβRI_ARG58:HH22–TβRII_ASP118:OD2	98.6	0.277 ± 0.010
TβRI_ARG58:HH12–TβRII_ASP118:OD1	97	0.282 ± 0.011
TβRI_ARG58:HH11–TβRII_PRO25:O	95.9	0.285 ± 0.011
TβRII_VAL22:H–TβRI_CYS76:O	91.2	0.298 ± 0.015
TβRI_SER66:HG–TβRII_ASP118:O	46.7	0.273 ± 0.013

**Table 3:** Relative binding free energy analysis of TB1/TB2 with TβRII and TβRI–TβRII complexes.

System (kcal/mol)		Van Der Waals Energy (ΔE_vdw_)	Electrostatic Energy (ΔE_ele_)	Polar Solvation Energy (ΔG_polar_)	Nonpolar Energy (ΔG_nonpolar_)	Binding Energy (ΔG_binding_)
TB1-TβRII		−54.05 ± 5.36	−30.29 ± 20.24	69.82 ± 17.52	−5.83 ± 0.5	−20.35 ± 7.89
TB2-TβRII		−44.85 ± 5.83	−304.11 ± 35.05	318.07 ± 30.90	−5.02 ± 0.43	−35.91 ± 9.29
TB1-TβRII-TβRI	TB1-TβRI	−0.18 ± 0.08	−2.82 ± 2.15	2.82 ± 2.13	−0.00 ± 0.01	−0.18 ± 0.11
	TβRI-TβRII	−36.87 ± 3.81	−81.96 ± 21.67	85.29 ± 19.92	−4.40 ± 0.21	−37.94 ± 4.06
	TβRI-(TB1-TβRII)	−37.05 ± 5.79	−84.78 ± 21.33	88.11 ± 19.59	−4.40 ± 0.21	−38.12 ± 4.07
TB2-TβRII-TβRI	TB2-TβRI	−29.29 ± 3.71	−4.09 ± 22.07	17.21 ± 20.40	−3.42 ± 0.34	−19.58 ± 4.00
	TβRI-TβRII	−46.37 ± 4.15	−200.01 ± 33.94	205.29 ± 30.72	−6.11 ± 0.30	−47.19 ± 5.94
	TβRI-(TB2-TβRII)	−76.12 ± 5.09	−205.01 ± 34.61	222.27 ± 31.76	−8.91 ± 0.35	−67.76 ± 7.70

**Table 4:** Energy decomposition of key residues in the TB2–TβRII–TβRI complex.

	Residue	Van der Waals (ΔE_vdw_)	Electrostatic (ΔE_ele_)	Polar Solvation (ΔG_polar_)	TOTAL (ΔG_total_)
TβRI	ARG:58	−4.67	−46.333	43.958	−7.045
	PHE:60	−5.804	−3.642	4.690	−4.756
	SER:66	−1.231	−8.091	7.031	−2.291
	LYS:67	−1.5	−31.932	32.92	−0.507
TβRII	VAL:22	−3.192	−2.367	2.16	−3.398
	PRO:25	−1.11	−8.781	5.926	−3.965
	ASP:118	0.01	−35.103	29.261	−5.833
	GLU:119	−1.006	−27.26	27.211	−1.055
TB2	ARG:10	−4.175	5.107	−0.769	0.163
	GLY:11	−1.937	−2.656	2.903	−1.69
	PRO:12	−3.076	25.292	−24.995	−2.779

## Data Availability

The original contributions presented in this study are included in the article. Further inquiries can be directed to the corresponding authors.
